# Trehalose alleviates the phenotype of Machado–Joseph disease mouse models

**DOI:** 10.1186/s12967-020-02302-2

**Published:** 2020-04-09

**Authors:** Magda M. Santana, Susana Paixão, Janete Cunha-Santos, Teresa Pereira Silva, Allyson Trevino-Garcia, Laetitia S. Gaspar, Clévio Nóbrega, Rui Jorge Nobre, Cláudia Cavadas, Hagar Greif, Luís Pereira de Almeida

**Affiliations:** 1 CNC - Center for Neuroscience and Cell Biology, University of Coimbra, Rua Larga, 3004-504 Coimbra, Portugal; 2 CIBB - Center for Innovative Biomedicine and Biotechnology, University of Coimbra, Coimbra, Portugal; 3 IIIUC - Institute for Interdisciplinary Research, University of Coimbra, Coimbra, Portugal; 4 Bioblast Pharma, Tel Aviv, Israel; 5 Faculty of Pharmacy, University of Coimbra, Coimbra, Portugal; 6 Present Address: Department of Biomedical Sciences and Medicine, Centre for Biomedical Research (CBMR), Algarve Biomedical Center (ABC), University of Algarve, Faro, Portugal

**Keywords:** Machado-Joseph disease, Spinocerebellar ataxia type 3, Polyglutamine disorder, Trehalose, Autophagy

## Abstract

**Background:**

Machado–Joseph disease (MJD), also known as spinocerebellar ataxia type 3, is the most common of the dominantly inherited ataxias worldwide and is characterized by mutant ataxin-3 aggregation and neuronal degeneration. There is no treatment available to block or delay disease progression. In this work we investigated whether trehalose, a natural occurring disaccharide widely used in food and cosmetic industry, would rescue biochemical, behavioral and neuropathological features of an in vitro and of a severe MJD transgenic mouse model.

**Methods:**

Two MJD animal models, a lentiviral based and a transgenic model, were orally treated with 2% trehalose solution for a period of 4 and 30 weeks, respectively. Motor behavior (rotarod, grip strength and footprint patterns) was evaluated at different time points and neuropathological features were evaluated upon in-life phase termination.

**Results:**

Trehalose-treated MJD mice equilibrated for a longer time in the rotarod apparatus and exhibited an improvement of ataxic gait in footprint analysis. Trehalose-mediated improvements in motor behaviour were associated with a reduction of the MJD-associated neuropathology, as MJD transgenic mice treated with trehalose presented preservation of cerebellar layers thickness and a decrease in the size of ataxin-3 aggregates in Purkinje cells. In agreement, an improvement of neuropathological features was also observed in the full length lentiviral-based mouse model of MJD submitted to 2% trehalose treatment.

**Conclusions:**

The present study suggests trehalose as a safety pharmacological strategy to counteract MJD-associated behavioural and neuropathological impairments.

## Background

Machado–Joseph disease or spinocerebellar ataxia type 3 (MJD) is a polyglutamine neurodegenerative disorder first described in people of Portuguese descent. MJD is considered the most common of the dominantly inherited ataxias worldwide [1], with the highest prevalence reported in Azorean islands (1:239 in Flores, Portugal) [2]. MJD is caused by an expansion of the CAG trinucleotide in the coding region of the *MJD1*/*ATXN3* gene, which is translated into a polyglutamine tract in the c-terminus of ataxin-3 protein. The mutation confers a toxic gain-of function to ataxin-3, with formation of neuronal intranuclear inclusions, neuronal dysfunction and degeneration [3–5]. Neuropathological alterations in MJD occur in the cerebellum, basal ganglia and brainstem and clinical features include progressive ataxia, ophtalmoplegia, dysarthria, dystonia, rigidity and distal muscle atrophies [1, 6, 7]. Progress towards understanding the pathogenesis of neurodegenerative disorders led to the identification of common pathological mechanisms and pathways in polyQ diseases that have become promising molecular targets for therapy. Over the last years, our group has focused on the study of autophagy in MJD and has identified this pathway as a relevant contributor to the neuropathology. We showed that autophagy is impaired in human brain tissue and fibroblasts of MJD patients and also in different animal and cellular models of MJD [8–11]. Moreover, upon local lentiviral-mediated expression of the autophagic protein 6/beclin-1 (Atg6/beclin-1) in the brain, we observed an alleviation of neuropathological and behavioral defects of MJD mouse models, evidencing that autophagy activation is a promising strategy to block MJD progression [8, 11]. However, these molecular approaches have limitations, particularly the risks associated to viral vector delivery and craniotomy. Moreover, the neuropathology of MJD involves multiple brain regions, so a strategy able to reach a broader distribution would be relevant. In an attempt to translate this strategy to the clinics in a short time-frame, we envisioned the systemic administration of a safe autophagy activator molecule as an advantageous alternative.

Trehalose, naturally present in several organisms, such as plants, yeasts, bacteria and invertebrates, is a non-reducing disaccharide, formed by two α-glucose molecules connected through an a,a-1,1 glycosidic linkage. It is currently used as a stabilizer in several food, cosmetic and pharmaceutical products, having an already proved safety profile [12, 13]. Trehalose has been reported to act as a natural autophagy inducer [14] and there is evidence of beneficial therapeutic effects of trehalose in relieving disease progression of protein aggregation diseases [15–20]. Nevertheless, no studies in MJD had yet been reported.

In this work, we designed a proof of concept study to assess whether trehalose alleviates behavioural and neuropathological phenotype features in MJD mouse models to evaluate the potential of this sugar to be used as a pharmacological drug to modify MJD disease progression.

## Materials and methods

### Drug

Trehalose was obtained from Pfanstiehl Inc (Illinois, USA) as trehalose dyhydrate, a white crystalline powder. In its solid form was stored at room temperature in a ventilated area. For in vitro and in vivo studies was prepared in water, as specified in the following sections.

### Cell culture and treatments

The mouse neuroblastoma cell line (Neuro-2A cells) was obtained from the American Type Culture Collection cell biology bank (CCL-131) and maintained in Dulbecco’s modified Eagle’s medium (complete medium; Gibco) supplemented with 10% fetal bovine serum, 100 U/ml penicillin and 100 U/ml streptomycin, at 37 °C in 5% CO_2_/air atmosphere. Neuro-2A transduction with lentiviral particles expressing mutant ataxin-3 72 CAG repeats (MutAtx-3) was performed as previously described [8]. Two weeks post-infection, cells were plated and treated with trehalose (1 mM, 10 mM or 100 mM) or control (vehicle) for 0.5 h, 1 h, 3 h, 6 h, 24 h, 48 h and 72 h. Treatments started 24 h after platting and medium was changed after 48 h. Trehalose incubations were repeated every 24 h and cells from all conditions were collected 72 h after platting.

### Resazurin assay

Cells were incubated with 0.1 mg/mL resazurin solution, diluted 1:10 in DMEM culture medium, for 45 to 60 min at 37 °C. From each well, 100 µl triplicates were placed into a 96-well plate and the absorbance was read at wavelengths of 570 nm and 600 nm. The absorbance ratio 570/600 nm was calculated and expressed as percentage of control.

### RNA extraction and RT-qPCR

Total RNA was extracted by using the NucleoSpin^®^ RNA extraction kit, according to manufacturer’s instructions (Macherey–Nagel). RNA was eluted in 40 µl of nuclease-free water and total RNA was quantified by optical density (OD) using a Nanodrop 2000 Spectrophotometer (Thermo Scientific). RNA purity was evaluated by measuring the ratio of OD at 260 and 280 nm. 1000 ng of total RNA were treated with DNase (Qiagen) and then reverse transcribed into double stranded cDNA by using the iScript cDNA Synthesis Kit^®^ (BioRad). cDNA samples were stored at − 20 °C until use.

Real-time PCR was performed using a standard SYBR-Green^®^ PCR kit protocol on a StepOne^®^ Detection System (Life Technologies). RT-PCR was carried out in 10 µL reaction, which included cDNA product (diluted 1:10), 1× SsoAdvanced SYBR Green Supermix and 0.5 µM of forward and reverse primers. Primers for SIRT1, LC3B, p62 and Beclin-1 were pre-designed and validated by QIAGEN (QuantiTect Primers, QIAGEN). Primers for mutant ataxin-3 and HPRT-1 were designed and validated by our group. Reverse-transcription and non-template controls were run in parallel. All reactions were performed in duplicate, according to the manufacturer’s recommendations: 95 °C for 30 s, followed by 45 cycles at 95 °C for 5 s and 60 °C for 30 s. HPRT-1 was used as reference gene. The mRNA fold increase or fold decrease with respect to control samples was determined by the delta-delta Ct method.

### Animals and experimental groups

#### Transgenic mouse model

A colony of MJD transgenic mice (MJDTg; C57BL/6 background) expressing the N-terminal-truncated human ataxin-3 with 69 glutamine repeats and a N-terminal hemagglutinin (HA) epitope, driven specifically in cerebellar Purkinje cells by the L7 promoter [21], was maintained at CNC animal facility by backcrossing heterozygous males with C57BL/6 females. For this experiment, 14 MJD transgenic female mice were weaned and genotyped at 4 weeks of age. Females have a less aggressive phenotype than males (unpublished data) and were therefore used in this experiment to avoid the use of weakened animals that could reach the humane critical endpoints over the long course of the experiment. Animals were housed in groups (2–5 per cage, depending on cohort study) in plastic cages (365 × 207 × 140 mm) with food and water ad libitum, and maintained on a 12-h light/dark cycle at a room with constant temperature (22 ± 2 °C) and humidity (55 ± 15%). The animals were allowed 1 week of acclimation to the surroundings before the beginning of the behavioral tests. Physical state of animals was evaluated daily and weight measured every week.

MJD Tg mice from different progenitors were randomly distributed into control and treatment group (7 animals/group) and then tested for behavior background before beginning the treatment. Trehalose was orally administered diluted in drinking distilled water at a final concentration of 2% (w/v) to the treatment group, from week 5 to week 35 of age. The control group was treated with vehicle (distilled water). A fresh solution of 2% Trehalose in water was prepared and changed twice a week until euthanasia of the animals.

#### Lentiviral-based mouse model

Twenty-five C57/Bl6 mice (Charles River, France), males, 8 weeks old, were housed in groups of 6 per cage, in plastic cages (365 × 207 × 140 mm) with food and water ad libitum, and maintained on a 12-h light/dark cycle at a room with constant temperature (22 ± 2 °C) and humidity (55 ± 15%). Males were used in this experiment to ensure comparability with previous studies using this model. The animals were stereotaxically-injected in the striatum with lentiviral vectors encoding mutant human ataxin-3 with 72 CAG repeats, as previously described [22]. After recovery from surgery, mice were randomly distributed into control (n = 13) and treatment groups (n = 12). The treatment group was orally-administered with trehalose diluted in drinking water at a final concentration of 2% (w/v) and the control group was treated with the vehicle (water). After 2 and 4 weeks of drug administration, mice were euthanized and brains collected for analysis (western bot and immunohistochemistry, respectively).

### Behavioral testing

Mice were trained on a battery of motor tests starting at 4 weeks of age (P21–25) and tested for behavioural background at 5 weeks of age (before the beginning of trehalose treatment, t = 0). Behaviour was then evaluated at different time points (t = 2, t = 6, t = 11, t = 14, t = 17, t = 20, t = 24 and t = 28 weeks of treatment) by an experienced operator in a blind fashion way. All tests were performed in the same dark room after at least 60 min of acclimatization.

#### Rotarod performance test

Motor coordination and balance were evaluated in a rotarod apparatus (Letica Scientific Instruments, Panlab, Barcelona, Spain). Mice were placed on the rotarod at a constant speed (5 rpm) and at accelerated speed (4 to 40 rpm in 5 min) and the latency to fall was recorded for a maximum of 5 min. Mice were allowed to perform four trials for each test and time point, with at least 30 min rest between trials. For analysis, the mean latency to fall off the rotarod of 4 trials was used.

#### Grip strength test

A grip strength test was performed to assess mice neuromuscular function. The grip strength of forelimbs was determined using a device consisted of a 300-g metal grid with a scale on. The animal was hung with its forepaws on the grid. The strength was determined as the weight pulled (g) from the scale. The test was performed during 10–15 consecutive trials and the mean of four best performances was taken to analysis. Mice body weight was used as a normalization factor.

#### Footprint analysis

Footprint analysis was performed at 28 weeks post-initiation of treatment. To obtain footprint patterns, mice front and forefeet were coated with blue and red non-toxic paints, respectively. Mice were allowed to walk on a blank greenish paper along a 100 cm long, 10 cm wide runway (with 15 cm high walls). Stride length was measured as the average distance of forward movement between each stride. Frontbase width and hindbase width were measured as the average distance between left and right front and hind footprints, respectively. These values were determined by measuring the perpendicular distance of a given step to a line connecting its opposite preceding and proceeding steps. The distance from left or right front footprint/hind footprint overlap was measured to evaluate uniformity of step alternation. A sequence of five consecutive steps was chosen for evaluation, excluding footprints made at the beginning and at the end of the run. Measurements were all made by the same operator. The mean value of each set of five was considered for each animal.

### Brain tissue collection

For histological analysis, the animals were given an avertin overdose (2.5 × 200 mg/g, i.p.) and were transcardially perfused with a phosphate solution (0.1 M) followed by fixation with 4% paraformaldehyde (PFA; Fluka, Sigma, Buchs, Switzerland). The brains were removed, post-fixed in 4% PFA for 24 h at 4 °C, and then cryoprotected by immersion in 25% sucrose/phosphate buffer for 48 h at 4 °C. The brains were frozen at − 80 °C and then the entire cerebellum (transgenic model) or brain (lentiviral-based model) was sliced into 30-μm midsagittal or 25-μm coronal sections, respectively, using a cryostat (LEICA CM3050S, Leica Microsystems) at − 21 °C. Sections were collected in anatomical series and stored at 4 °C as free-floating sections in phosphate buffered saline (PBS) supplemented with 0.05 mM sodium azide until processing.

For western blot analysis (lentiviral-based model), animals were euthanized by cervical dislocation. Tissue punches from striatum were collected and keep at -80 °C until use.

### Histological analysis of brain tissue

#### Cresyl violet staining

Eight sagittal sections along the extent of the mice left hemicerebellum (inter-section distance of 240 µm, which corresponds to eight sections of 30 µm) were mounted in gelatin covered microscope slides and dried at room temperature. Sections were then stained with cresyl violet for 5 min, differentiated in 70% ethanol, dehydrated by passing through 95% ethanol, 100% ethanol and xylene solutions and mounted with Eukitt (Sigma-Aldrich).

#### Immunofluorescent staining

Immunofluorescent staining was performed in eight sagittal sections over the extent of the mice left hemicerebellum (inter-section distance of 240 µm, which corresponds to eight sections of 30 µm). Free-floating sections were washed with PBS 0.1 M and blocked for 1 h at room temperature in 0.3% Triton X-100 in PBS 0.1 M supplemented with 10% normal goat serum (NGS; Gibco). Sections were then incubated with the following primary antibodies: mouse monoclonal anti-HA antibody (1:1000; InvivoGen, San Diego, CA, USA) and rabbit polyclonal anti-calbindin-28 K antibody (1:1000; Merck Millipore) overnight at 4 °C. After incubation, sections were washed three times with PBS and then incubated with the corresponding secondary antibody goat anti-mouse conjugated to the fluorophore 488 (1:200; Molecular Probes, Oregon, USA), diluted in blocking solution, for 2 h, at room temperature. Finally, sections were washed three times in PBS, counterstained with 4′,6-diamidino-2-phenylindole, washed again and mounted with Fluorsave (Calbiochem, Germany).

#### Immunohistochemistry

Immunohistochemistry was performed in twelve coronal sections covering the extent of the mice striata (25 μm-thick sections at 200 μm intervals). After the blockage of endogenous peroxidases with phenylhidrazyne/phosphate solution, free-floating sections were washed with PBS 0.1 M and blocked for 1 h at room temperature in blocking solution (0.1% Triton X-100 in PBS 0.1 M supplemented with 10% normal goat serum). Sections were processed overnight at 4 °C in blocking solution with the following primary antibodies: a polyclonal rabbit anti-ubiquitin antibody (1:300; Enzo Life Sciences) and a polyclonal rabbit anti–DARPP-32 antibody (1:1000; Merck Millipore), followed by 2-h incubation at room temperature with the respective biotinylated goat anti-mouse or anti-rabbit antibodies (1:200; Vector Laboratoires). Bound antibodies were visualized using the Vectastain ABC kit, with 3,30-diaminobenzidine tetrahydrochloride (DAB metal concentrate, Pierce) as substrate. Dry sections were mounted in gelatin-coated slides, dehydrated with ethanol solutions and xylene and mounted in Eukit (Sigma-Aldrich).

### Quantitative analysis of histological sections

#### Quantification of cerebellum volume (transgenic model)

Quantification was made over eight cresyl violet staining sagittal sections over the extent of the mice left hemicerebella in a blind fashion. Mosaic pictures of these sections were taken using a PALM Laser microdissection microscope (Carl Zeiss, Germany) with a 20× objective. Volume was assessed by measuring the area of the cerebellum in each section using Fiji software. Hemicerebellum final volume was extrapolated using the following formula: volume = (area × section thickness) × number of sections.

#### Quantification of cerebellum layers thickness (transgenic model)

Quantification was made over four cresyl violet-stained sagittal sections. Mosaic pictures were taken using a PALM Laser microdissection microscope (Carl Zeiss, Germany) with a 20× objective. For each section, layers length was blindly determined using Fiji software. Layers thickness was assessed by measuring the mean width of cerebellum layers (molecular + granular + Purkinje cell layers) at interlobular regions.

#### Quantification of Purkinje cells and mutant ataxin-3 aggregates (transgenic model)

Quantitative analysis of number of Purkinje cells (calbindin-positive cells) and mutant ataxin-3 aggregates (HA aggregates) was performed over eight sections. To calculate the number of calbindin-positive cells, mosaic pictures were taken using a PALM Laser microdissection microscope (Carl Zeiss, Germany) with a 20× objective. Images were visualized with ImageJ software and cells were then manually counted by an operator in a blind fashion. Total number (no) of purkinje cells per hemicerebellum was calculated by extrapolation, using the formula:

$${\text{no}}\;{\text{of}}\;{\text{purkinje}}\;{\text{cells}}\, = \,\left( {{\text{no}}\;{\text{of}}\;{\text{cells in section 1}}\, + \,{\text{no of cells in}}\;{\text{section}}\;2\, + \, \cdots \, + \,{\text{no of cells}}\;{\text{in}}\;{\text{section}} \; 8} \right) \times 8 {\text{ sections}}$$

Total number of HA aggregates was manually counted by an operator in a blind fashion by visualizing immunostained sections using a Axioskop 2 plus microscope (Carl Zeiss) and calculated by extrapolation, using the formula:

$${\text{no of mutant ataxin{-}3 aggregates}}\, = \,\left( {{\text{no of aggregates in section 1}}\, + \,{\text{no of aggregates in}}\;{\text{section 2}} + \cdots + {\text{no of aggregates in}}\;{\text{section 8}}} \right) \times 8 {\text{ sections}}$$

#### Quantification of mutant ataxin-3 aggregates size (transgenic model)

Quantitative analysis of mutant ataxin-3 aggregates size was performed over four sections. Three representative pictures of lobule IX were taken using a Cell Observer Spinning Disk (Carl Zeiss, Germany) with a 100× objective. Z projections were visualized and the diameter was manually determined by an operator in a blind fashion using Fiji software.

#### Quantification of DARPP-32 depleted volume, ubiquitin inclusions number and size (lentiviral model)

Quantification was made over twelve coronal sections per animal (25 μm-thick sections at 200 μm intervals) on the extent of the mouse striatum, in a blind fashion. Pictures of DARPP-32 stained sections were taken using a 5× objective using an Axioskop 2 Plus microscope (Carl Zeiss, Germany). Volume was assessed by measuring the area of the lesion in each section using Fiji software. Final volume was extrapolated as described above. Pictures of ubiquitin-stained sections were taken using a 20× objective, with the same microscope. The analyzed areas of the striatum encompassed the entire region containing ubiquitin aggregates. All inclusions were blindly automatically counted using Fiji software. Total number of ubiquitin aggregates was extrapolated as described for ataxin-3 aggregates. Data were normalized to the integrated lentivirus copy number, quantified as described below.

### Protein extraction and preparation

Cellular extracts were obtained by scrapping cells with radioimmunoprecipitation assay (RIPA) buffer solution [50 mM Tris·HCl, pH 7.4; 150 mM NaCl; 5 mM EDTA; 1% Triton X-100; 0.5% sodium deoxycholate; 0.1% sodium dodecyl sulfate (SDS)] supplemented with protease inhibitors (Roche), 200 μM phenylmethylsulphonylfluoride, 1 mM dithiothreitol (DTT), 1 mM Na_3_VO_4_ and 10 mM NaF.

Tissue extracts were lysed in RIPA buffer, supplemented as described above, by 2 series of 4 s ultra-sound pulse (1 pulse/s). Total protein lysates were stored at − 20 °C and protein concentration was quantified with BCA protein assay (Pierce Biotechnology, Thermo Scientific, USA).

### Western blotting

Protein samples were denaturated in SDS sample buffer (0.5 M Tris, 30% glycerol, 10% SDS, 0.6 M DTT, 0.012% bromophenol blue) for 5 min at 95 °C. Samples were then separated in a 4–12% SDS–polyacrylamide gel electrophoresis (SDS-PAGE). Ponceau S staining was performed after transference and the membranes were blocked with 5% non-fat milk in TBS-T (137 mM NaCl, 20 mM Tris, 0.1% Tween 20, pH 7.6) following by incubation overnight at 4 °C with mouse monoclonal anti-β-actin antibody (clone AC74;1:5000; Sigma-Aldrich), mouse monoclonal anti-β-tubulin antibody (clone SAP.4G5;1:10,000; Sigma-Aldrich), mouse anti-GAPDH (1:500; Merck Millipore), mouse monoclonal anti-HA antibody (1:1500; InvivoGen, San Diego, CA, USA), rabbit monoclonal anti-p62 antibody (1:1500; Cell Signaling Technology) and rabbit monoclonal anti-LC3B antibody (1:1000; Cell Signaling Technology) diluted in blocking solution or in 3% bovine serum albumin (for cell signaling antibodies). After three washes with TBS-T, the membranes were incubated for 1 h, at room temperature, with an alkaline phosphatase-linked secondary antibody, specific to rabbit or mouse immunoglobulin G (1:20,000, Amersam Biosciences, GE Healthcare, UK). Immunoreactive bands were visualized using enhanced chemifluorescence (ECF) substrate in the Versa-Doc 3000 imaging system (Bio-Rad, USA) and densitometry of the bands was quantified using ImageJ software. The specific optical density was normalized to the total protein, measured by ponceau S, or to the amount of β-actin [23].

### Integrated lentivirus copy number quantification

Twelve mounted histological slides were immersed in xylene for 2 days and then hydrated with ethanol solutions and water. Lentiviral-injected striata were removed with a scalpel and DNA extraction was performed using the GeneRead DNA FFPE Kit (Qiagen), starting from step 6 of manufacturer’s protocol. Purified cellular genomic DNA was then quantified by optical density (OD) using a Nanodrop 2000 Spectrophotometer (Thermo Scientific) and the purity was evaluated by measuring the ratio of OD at 260 and 280 nm. Copy number of integrated lentiviruses (proviruses) present in the transduced cells of striatum was detected by qPCR using the Lenti-X Provirus Quantitation Kit (Takara) and according to the manufacturer’s instructions. Briefly, serial dilutions of the cellular gDNA were subjected to qPCR amplification alongside dilutions of a calibrated Provirus control template. The final result was expressed in terms of provirus copies (vg)/cell.

### Statistical analysis

Raw data analysis was conducted using Prism GraphPad software. Outliers were excluded from analysis using Grubb’s test. For behavior results, mean values for each animal were calculated and a two-tailed Student’s *t* test (footprint analysis) or a trend analysis to compare linear regression slopes using the two-tailed *t* test hypothesis (other behavioral tests) was performed. For other analysis, statistics was performed using the two-tailed Student’s *t* test. Data were represented as mean ± SEM.

## Results

### Trehalose activates autophagy and reduces mutant ataxin-3 protein levels in neuro-2a cells expressing mutant ataxin-3

**Fig. 1:**
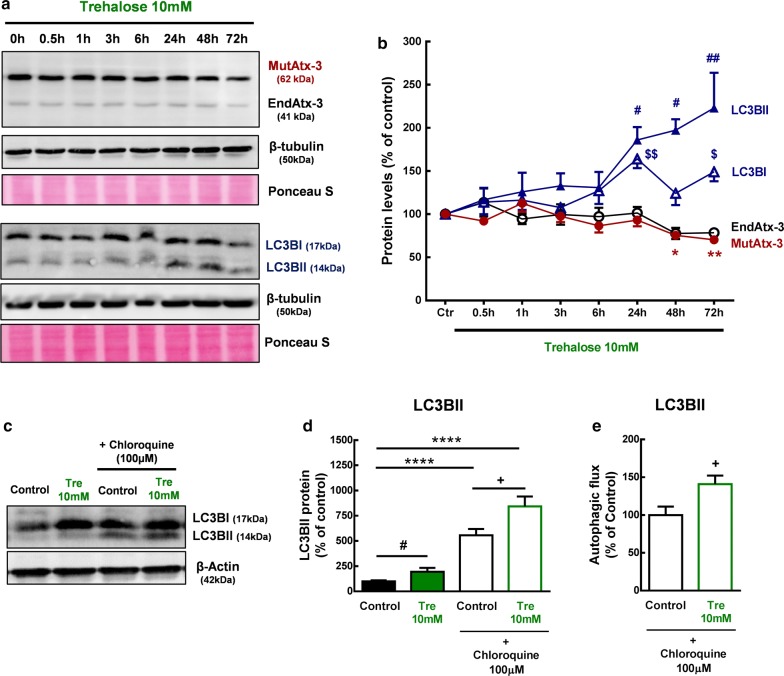
Autophagy activation by trehalose in neuro-2A cells expressing mutant ataxin-3. Neuro-2A cells expressing mutant ataxin 3 (MutAtx-3) were treated with 10 mM trehalose or vehicle (control) from 0.5 to 72 h (h). Protein levels of LC3BI, LC3BII, endogenous ataxin-3 (EndAtx-3) and mutant ataxin-3 (MutAtx-3) were quantified by western blot. **a** Representative picture of Western blot. **b** Quantitative densitometric analysis of western blot bands. Values are presented as mean ± SEM (n = 4–5). Results are expressed as % of control. *^,#, $^p < 0.05; **^,##, $$^p < 0.01, compared to control. One-way analysis of variance (ANOVA), followed by a Dunnett’s post hoc analysis for multiple comparisons; outliers were excluded using the Grubb’s test. Cells were then incubated for 72 h with trehalose 10 mM, in the presence and absence of the lysosomal degradation inhibitor, chloroquine, for 6 h before protein collection. Protein levels of LC3BII were quantified by western blot. **c** Representative picture of Western blot. **d** Quantitative densitometric analysis of western blot bands. Values are presented as mean ± SEM (n = 6). Results are expressed as % of control. ^##^p < 0.01, compared to control; Student’s *t* test. ****p < 0.0001, compared to control; One-way analysis of variance (ANOVA), followed by a Dunnett’s post hoc analysis for multiple comparisons. **e** Autophagic flux quantification by the LC3BIIturnover assay. Difference between densitometric intensity of LC3II bands between control and trehalose conditions in the presence and absence of lysosomal inhibitors. Values are presented as mean ± SEM (n = 6). ^+^p<0.05, compared to control plus chloroquine; Student’s *t* test

We previously showed that autophagy is compromised in MJD and that its activation can ameliorate the disease neuropathology [8, 11]. To investigate the ability of trehalose to increase autophagy in MJD we used Neuro 2A cells expressing the human isoform of MutAtx3, a previously described in vitro model of MJD [8]. We first observed that trehalose could be used to treat these cells up to 10 mM concentration for a period of 72 h without changing metabolic activity (Additional file 1: Fig. S1). Cells were then incubated with 10 mM trehalose for 0.5, 1, 3, 6, 24, 48 and 72 h, and the levels of the transient autophagosomal membrane-bound form of LC3B (LC3BI and LC3BII), a marker of the autophagic process, were measured by western blot. As shown in Fig. 1a, b, LC3BII protein levels significantly increased after 24 h, 48 h and 72 h treatment with 10 mM trehalose, when compared to control. More relevant, this effect was accompanied by a significant decrease in protein levels of mutant ataxin-3 (Fig. 1a, b; Additional file 2: Fig. S2 and Additional file 3: Fig. S3). Transcript levels of mutant ataxin-3 and autophagy markers (LC3B, p62 and beclin-1) did not change after 10 mM trehalose treatment for a period of 72 h (Additional file 4: Fig. S4).

Increased levels of LC3BII do not necessarily indicate an increase in total autophagic flux as it can also be a consequence of impaired autophagosome-lysosome fusion [24]. LC3BII is degraded at the final stages of autophagy and when lysosomal degradation is blocked there is an accumulation of this protein. Accumulation of this protein upon lysosomal degradation inhibition is indicative of increased autophagic flux. To confirm whether trehalose was indeed increasing autophagy, we evaluated LC3BII protein levels in the presence and absence of chloroquine, an inhibitor of lysosomal degradation. Again, an increase in LC3BII protein levels was observed in N2A cells expressing MutAtx-3, after 72 h treatment with 10 mM trehalose, as compared with control condition (Fig. 1c, d; ^##^p < 0.01). Moreover, upon lysosomal degradation inhibition with chloroquine 100 µM for 6 h, an accumulation of LC3BII was observed for both control and trehalose conditions, respectively (Fig. 1d; ****p < 0.0001, compared to control). More important, an increased accumulation of LC3BII was observed in 10 mM trehalose plus chloroquine condition, when compared to control plus chloroquine (Fig. 1d; ^+^p<0.05, compared to control), further indicating that trehalose increased autophagic flux. We next determined autophagic flux by the LC3BII turnover assay, which compares the LC3II protein levels between control and trehalose conditions in the presence and absence of lysosomal inhibitors (LC3BII net flux) [25]. As shown in Fig. 1e, 10 mM trehalose increased LC3BII autophagic flux in neuro-2A cells expressing mutant ataxin-3, indicating an activation of autophagy. Altogether, these data shows that trehalose activates autophagy and reduces mutant ataxin-3 levels in this in vitro model of MJD.

### Trehalose alleviates motor deficits in MJD transgenic mice

**Fig. 2:**
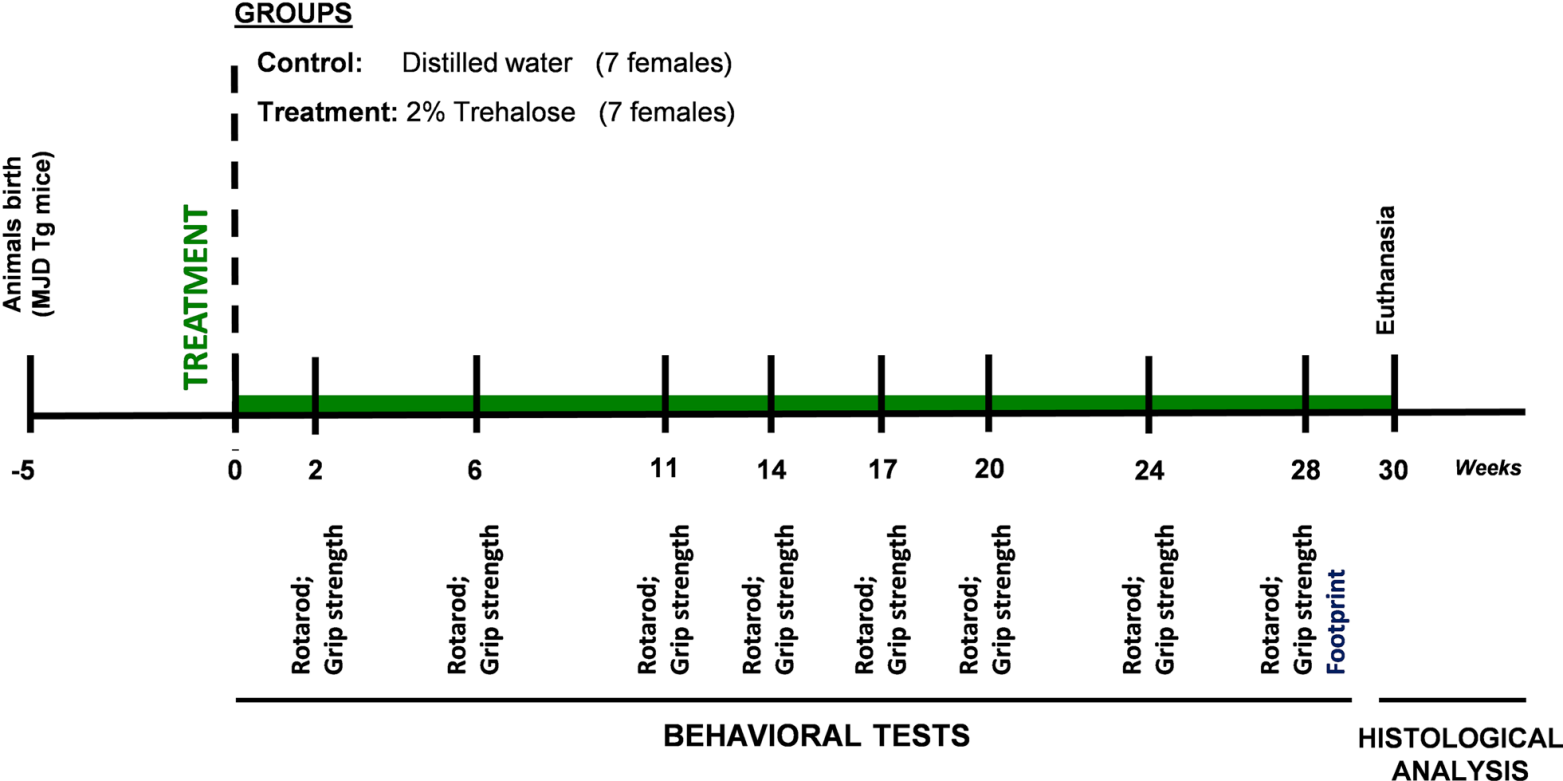
Study design for the transgenic MJD model. Fourteen MJD transgenic female mice were weaned and genotyped at 4 weeks old. MJD transgenic mice were then randomly distributed into Treatment and Control groups that received either 2% Trehalose in drinking water or fresh distilled water, respectively, from 5 weeks up to 35 weeks of age. Motor and neuromuscular function of the animals was evaluated by performing rotarod (constant and accelerated speed) and grip strength tests, respectively, before the beginning of Trehalose treatment (5 weeks of age; t = 0) and at several time points (t = 2, t = 6, t = 11, t = 14, t = 17, t = 20, t = 24, t = 28 weeks of treatment). The footprint analysis was performed only at t = 28 weeks of treatment. The animals were sacrificed at 35 weeks of age (t = 30) and brain tissues were collected for histopathological analysis of the cerebellum

Building on the obtained in vitro results, we designed an in vivo experiment to investigate whether administration of trehalose to a transgenic mouse model of MJD would alleviate behavioral and neuropathological defects. Fourteen MJD transgenic female mice were distributed into control and treatment groups and a 2% trehalose solution was orally administered to the treatment group over a period of 30 weeks (Fig. 2). Dose was chosen accordingly to previous studies in animal models of protein aggregation diseases [17, 20]. Mice body weight and general physical health was evaluated each week until euthanasia of animals. Throughout the whole study, trehalose had no effect in body weight and caused no apparent impact in mice general health (Additional file 5: Fig. S5).

**Fig. 3:**
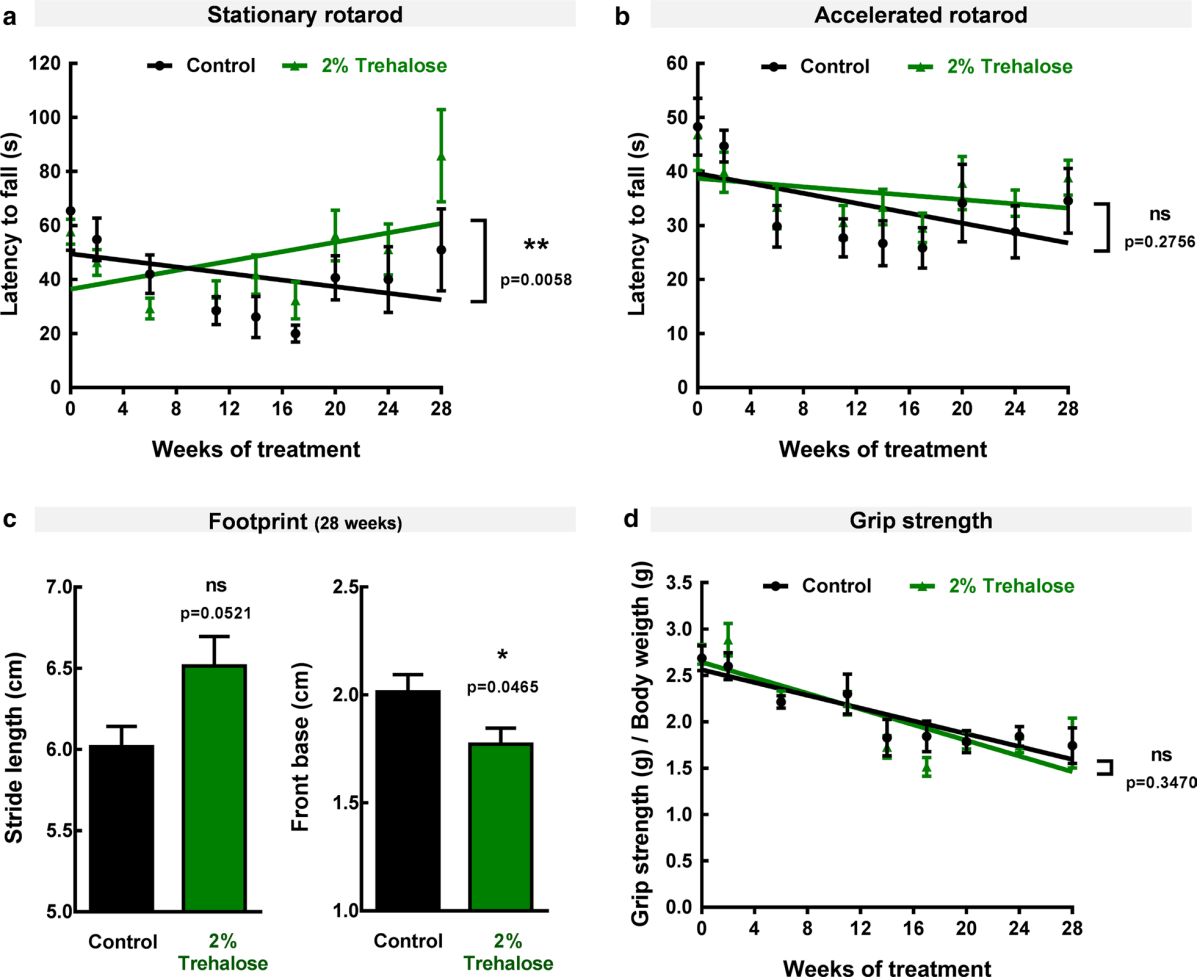
Motor and neuromuscular function of MJD transgenic mice treated with 2% Trehalose. MJD transgenic females were randomly distributed into control (n = 7; black) and treatment groups (n = 7; green) and orally treated either with vehicle or 2% Trehalose. Rotarod and grip strength tests were done before the beginning of the treatment and at different time points until 28 weeks (**a**, **b**, **d**). Statistical analysis was performed comparing linear regression slopes by a two-tailed *t* test hypothesis. In rotarod tests, 2% Trehalose increased the latency time to fall of MJD transgenic females (**a**, **b**). No statistically significant differences in forelimb strength were observed between control and 2% Trehalose groups (**d**). Additionally, footprint patterns were evaluated 28 weeks after treatment (**c**). Statistical analysis for footprint was performed by using the Student’s *t* test hypothesis. 2% Trehalose reduced gait deficits of MJD transgenic females, increasing the stride length and decreasing the frontbase distance (**c**). Data are presented as mean ± SEM, * < 0.05; ** < 0.01; ns = not significant, comparing to controls

To evaluate the therapeutic potential of trehalose in recovering the balance and motor function deficits of MJD transgenic mice, we performed the rotarod test. Behavioural testing evaluation started before the beginning of the treatment (5 weeks old; t = 0 weeks of treatment) and was repeated at different time points until 28 weeks of trehalose administration (Fig. 2). A linear regression analysis was used to assess the time course of behavioral performance [26]. At 5 weeks of age, MJD transgenic mice already present a marked phenotype characterized by ataxic movement and difficulty to walk and equilibrate, as previously described [9, 21, 27]. Trehalose administration improved motor performance of MJD transgenic females, as shown by the increase in time that treated females equilibrated in rotarod apparatus, compared to control animals, which over time exhibited a decrease in the latency time to fall (Fig. 3a, b). This effect was statistically significant for stationary rotarod performance test [*Linear regression slopes for stationary rotarod:* Control = − 0.6082 ± 0.3820, 2% trehalose = 0.8690 ± 0.3620, p = 0.0058, (Fig. 3a); *Linear regression slopes for accelerated rotarod:* Control = − 0.4599 ± 0.1863, 2% trehalose = − 0.1966 ± 0.1519; p = 0.2756).

MJD transgenic mice also exhibit significant gait deficits, presenting reduced stride length and enlarged front and hindbase, which can be evaluated by analysis of footprint patterns [11]. Therefore, at 28 weeks of treatment, a footprint analysis was additionally performed to investigate whether trehalose could rescue limb and gait ataxia of MJD transgenic mice. As shown in Fig. 3c a close to significant increase in the stride length (Control = 6.02 ± 0.12 cm, 2% trehalose = 6.51 ± 0.18 cm, p = 0.052, Student’s *t* test) and a significant decrease in the front base width (Control = 2.01 ± 0.08 cm, 2% trehalose = 1.77 ± 0.07 cm, p = 0.047; Student’s *t* test) was observed in MJD transgenic females treated with 2% trehalose, revealing that trehalose reduced the gait deficits of MJD transgenic females. Overall, behavioural data show that 2% trehalose treatment alleviates motor and coordination deficits in MJD transgenic mice.

The neuromuscular function is also affected in MJD patients, since the primary function of the cerebellum is to maintain the excitability of the motor cortex and the subsequent control of movement [28]. Such as in clinical observations in patients, MJD transgenic mice display significant strength deficit [22]. Therefore, we performed the grip strength test to assess the ability of trehalose in recovering neuromuscular deficits, but no statistically significant differences in forelimb strength were observed between groups (*Linear regression slopes:* Control = − 0.0345 ± 0.0057, 2% trehalose = − 0.0422 ± 0.0058; p = 0.3470), suggesting that trehalose had no effect in recovering neuromuscular function of this MJD transgenic mouse model.

### Trehalose reduces cerebellar atrophy and mutant ataxin-3 aggregate size in Purkinje cells of Machado–Joseph disease transgenic mice

**Fig. 4:**
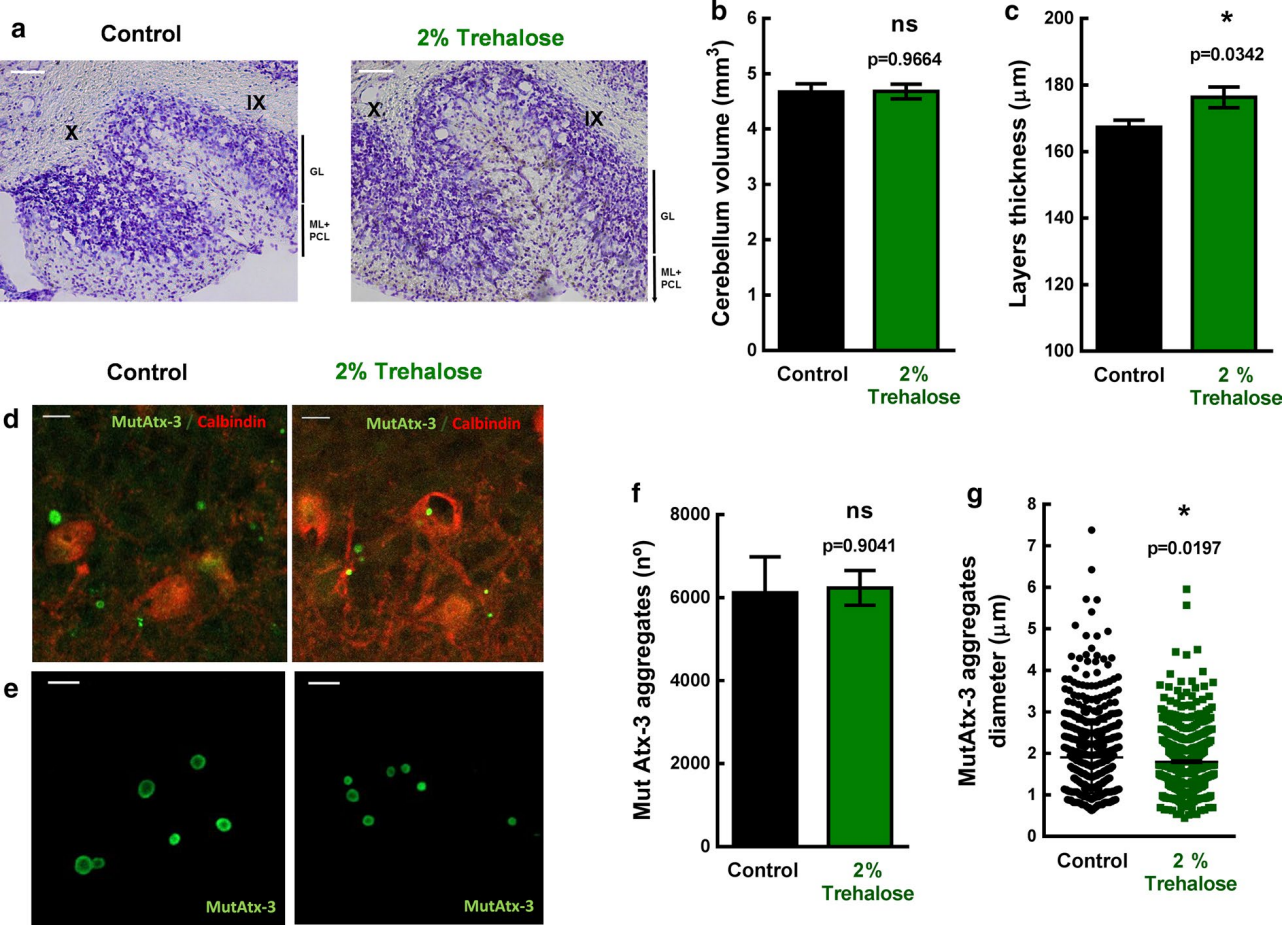
Neuropathological analysis of brain tissue from MJD transgenic mice treated with 2% Trehalose. Cresyl violet staining was performed on left hemicerebellum sections from MJD transgenic female mice treated with distilled water (control; n = 7) or 2% Trehalose (n = 7) for 30 weeks. **a** Representative picture of cerebellum layers from control and 2% Trehalose-treated MJD transgenic mice. Abbreviations: GL, Granular layer; ML, Molecular layer; PCL, Purkinje cell layer. Scale = 50 µm. **b**, **c** Trehalose has no effect in cerebellum volume, but increased the thickness of cerebellum layers compared to controls. Data are presented as mean ± SEM. Statistical analysis was performed using the two-tailed Student’s *t* test. * < 0.05, comparing to controls; ns = not significant. Immunofluorescent staining was also performed on left hemicerebellum sections from MJD transgenic mice treated with distilled water (control; n = 7) or 2% Trehalose (n = 7) for 30 weeks. **d** Representative confocal image of immunofluorescent staining against HA (MutAtx-3; green) and calbindin (red) of left hemicerebellum sections from MJD transgenic mice treated with distilled water (control; n = 7) or 2% Trehalose (n = 7) for 30 weeks. Scale bar = 10 µm. **e** Representative picture of aggregates size from control and 2% Trehalose-treated MJD transgenic mice. Scale bar = 5 µm. **f**, **g** The total number of aggregates and aggregates size (diameter) in a representative lobule (lobule IX) were quantified. Trehalose had no effect in total number of aggregates, but decreased aggregates diameter in lobule IX. Data are presented as mean ± SEM. Statistical analysis was performed using the two-tailed Student’s t test. *ns* not significant

Cerebellar atrophy is an important neuropathological hallmark of this MJD animal model [11, 21], as it is typically present in MJD patients [29]. To evaluate if the phenotypic improvement correlated with preservation of the cerebellar neuroanatomy we performed the Nissl staining with cresyl violet, which is a classical histological method widely used to study the cytoarchitecture of brain areas [30, 31]. The total cerebellar volume and cerebellar layers thickness were measured. Despite no observation of differences between groups for cerebellar volume (Fig. 4b; Control = 4.67 ± 0.15 mm^3^; 2% trehalose = 4.68 ± 0.13 mm^3^; p = 0.9664), cresyl violet staining revealed larger thickness (Fig. 4a, b; Control = 167.3 ± 2.1 µm, 2% trehalose = 176.3 ± 3.1 µm, p = 0.0342) of cerebellar layers of treated MJD transgenic mice, when compared to controls (Fig. 4c), suggesting prevention of neurodegeneration by trehalose.

Mutant ataxin-3 has toxic properties due to the expanded polyQ tract, aggregating and accumulating in neuronal cells [3, 4]. As shown in Fig. 4d, and previously described [9, 21], neuronal inclusions accumulate in Purkinje cells of the cerebellar cortex in this transgenic mouse model. Purkinje cells were stained against calbindin-28 K, commonly used as Purkinje cell marker, but a very weak and diffuse immunofluorescent signal was observed in mice at this age (35 weeks old), particularly in controls (Fig. 4d). This loss of calbindin staining is associated with neuronal dysfunction of Purkinje cells [32]. As can be observed, Purkinje cells presented with irregular cell bodies and a disordered dendritic network. When treated with 2% trehalose, an improvement of the immunofluorescent pattern was observed, and Purkinje cellular bodies were less irregular and the dendritic network more preserved (Fig. 4d).

We then investigated whether trehalose could modify the number and the size of mutant ataxin-3 aggregates in the cerebellum of MJD transgenic mice. This mouse model expresses a NH2-truncated form of the ataxin-3 protein (with Q69), that lacks the 286 NH2-terminal amino acid residues, but is tagged by an HA epitope [21], so an immunostaining for the HA tag was performed to measure aggregation. The total number of mutant ataxin-3 aggregates in the whole cerebellum was counted but no differences were observed between 2% trehalose and control MJD transgenic mice (Fig. 4f, Control = 6115 ± 865; 2% trehalose = 6233 ± 419; p = 0.9041). However, we observed that trehalose significantly decreased the diameter of mutant ataxin-3 aggregates in a representative lobule (lobule IX) of the cerebellum (Control = 1.91 ± 0.03 µm; 2% trehalose = 1.80 ± 0.03 µm; p = 0.0197; Fig. 4e, g). These results show that trehalose alleviates cerebellar neuropathology of this transgenic mouse model of MJD.

### Trehalose ameliorates neuropathology of a lentiviral-based mouse model of MJD

**Fig. 5:**
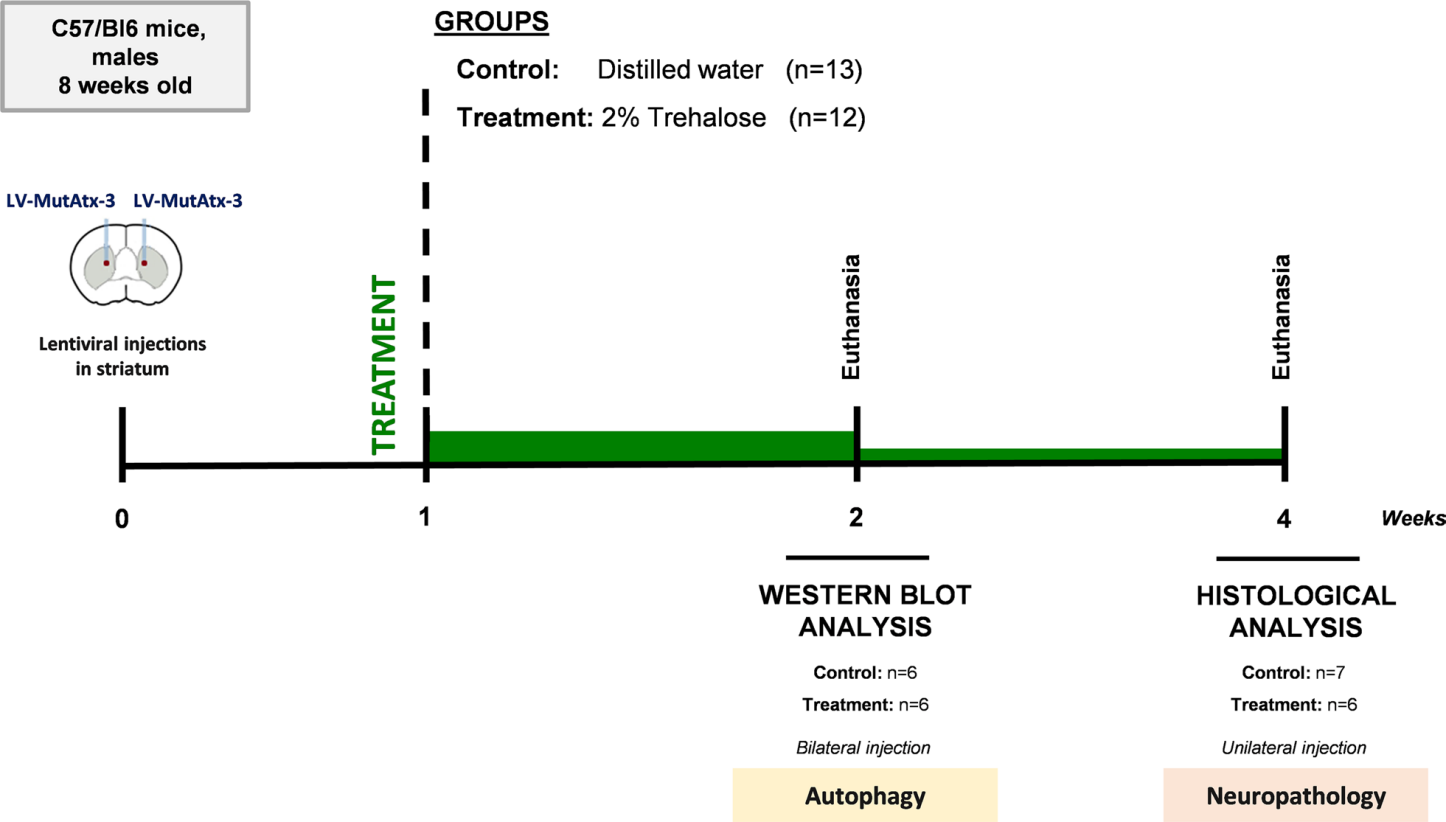
Study design for the lentiviral based MJD model. Twenty-five C57/Bl6 mice, males, 8 weeks old, were stereotaxically-injected in the striatum with lentiviral vectors encoding mutant human ataxin-3 with 72 CAG repeats. Mice were then randomly distributed into treatment (n = 12) and control groups (n = 13) that received either 2% Trehalose in drinking water or fresh distilled water, respectively, After 2 and 4 weeks of drug administration, mice were euthanized and brains collected for analysis

**Fig. 6:**
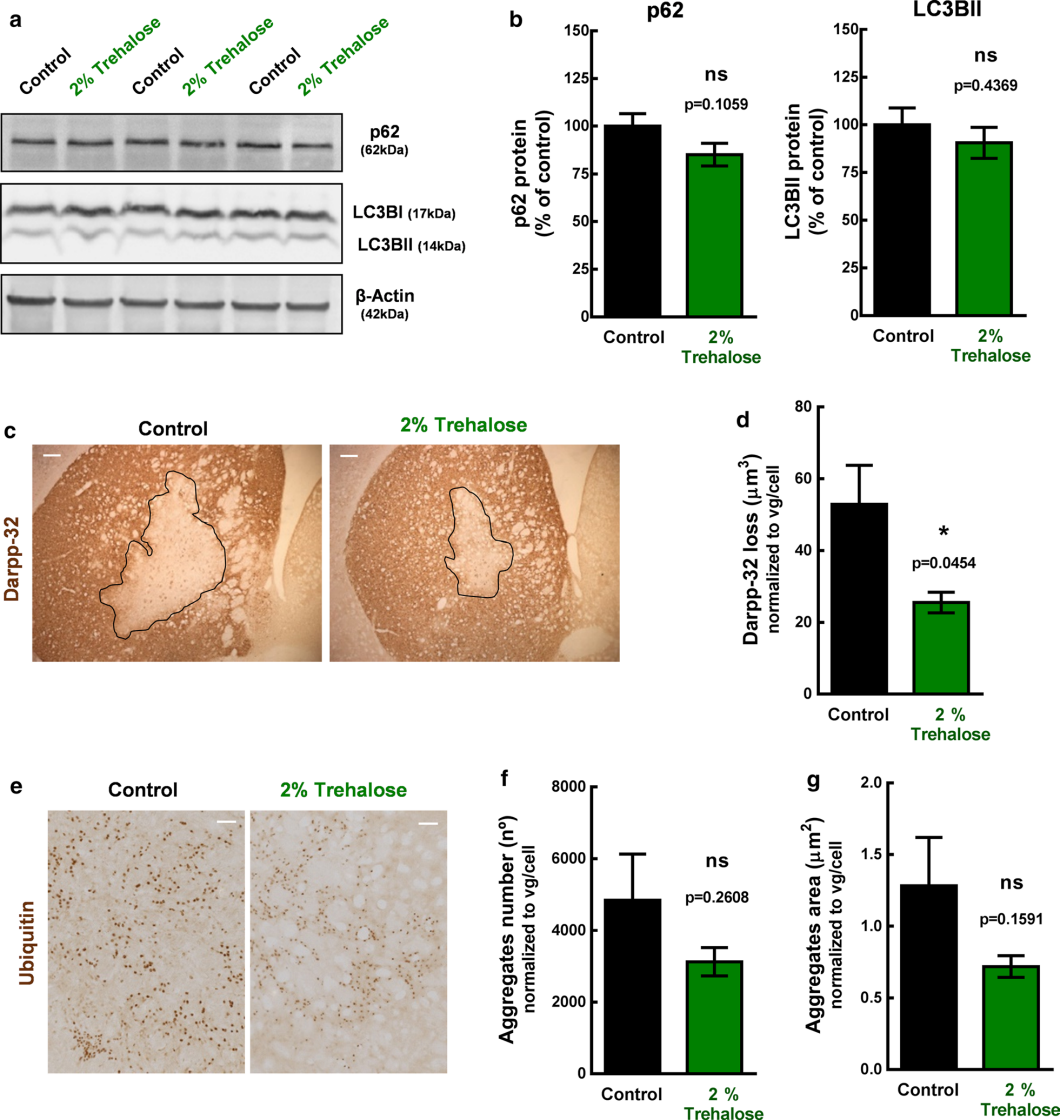
Brain tissue analysis of the lentiviral-based mouse model treated with 2% Trehalose. C57/Bl6 mice, males, 8-weeks old, were stereotaxically-injected bilaterally in the striatum with lentiviral vectors encoding mutant human ataxin-3 with 72 CAG repeats and distributed into control and treatment groups that received either water or 2% Trehalose, respectively, during 2 weeks. Protein levels of autophagy markers were quantified by western blot in both left and right striatal punches. **a** Picture of western blot membranes. **b** Quantitative densitometric analysis of autophagy markers (p62 and LC3BII) western blot bands. Values are presented as mean ± SEM (n = 12–13 striatum per group from 6 to 7 animals). Student’s *t* test, compared to control. Loss of darpp-32 staining and ubiqutitin aggregates were evaluated by immunohistochemistry, after 4 weeks of 2% trehalose administration, in mice stereotaxically-injected unilaterally in the striatum with mutant human ataxin-3 encoded lentiviral vectors. **c** Representative figure of darpp-32 immunohistological staining. Scale = 100 µm. **d** The darpp-32 depleted immunoreactivity was decreased in mice treated with 2% trehalose. Data are presented as mean ± SEM. Statistical analysis was performed using the two-tailed Student’s *t* test. *p < 0.05, compared to control. **e** Representative picture of ubiquitin staining. Scale bar = 50 µm. **f**, **g** Trehalose had no statistically significant effect in total aggregates number and size, but a tendency for decrease was observed. Data are presented as mean ± SEM. Statistical analysis was performed using the two-tailed Student’s *t* test. ns = not significant

To further strengthen the results obtained with the transgenic model, we used a striatal lentiviral-based mouse model of MJD to evaluate whether trehalose could alleviate neuropathology. In this model, the neuropathological features of MJD are induced by the injection of lentiviral vectors encoding a full-length human mutant ataxin-3 carrying 72 CAG repeats [22]. Twenty-five mice were injected in the striatum with the lentiviral vectors and distributed into control (n = 13) and treatment groups (n = 12), which were orally administered with water and 2% trehalose solution, respectively. Accordingly to previously studies from our group [22, 33], we expected to observe a positive effect of the treatment after 4 weeks administration, which would rationally be anticipated by an increase in autophagy. Thus, half of the animals (6 controls and 6 treated mice) were sacrificed after 2 weeks to evaluate autophagy markers and the other half (7 controls and 6 treated mice) after 4 weeks to evaluate neuropathology, by western blot and immunohistochemistry, respectively (Fig. 5). After 2 weeks administration of 2% trehalose, no statistically significant differences in LC3BII and p62 protein levels were detected between groups (Fig. 6a, b, (Additional file 7: Fig. S7). At this time point, levels of mutant ataxin-3 (aggregates, oligomers and soluble form) were also similar in animals administered with water and 2% trehalose (Additional file 6: Fig. S6).

Histologically, this mouse model is characterized by the presence of ubiquitinated aggregates and the loss of Darpp-32 immunoreactivity [22, 33]. Darpp-32 is a protein of the dopaminergic signaling pathway and loss of its immunoreactivity is indicative of early neuronal dysfunction. The darpp-32-depleted area in histological sections is thus used to quantify the neuropathological defects induced by mutant ataxin-3 expression. After 4-weeks administration of 2% trehalose, treated mice had a smaller lesion in the striatum, when compared to control animals (Area_Control_ = 52.89 ± 10.88 µm^2^/vg/cell; Area_Treatment_ = 25.56 ± 2.87 µm^2^/vg/cell; p = 0.0454; Fig. 6c, d). Despite no statistically-significant differences between groups regarding the aggregate number (Control = 4842 ± 1290; 2% trehalose = 3125 ± 395; p = 0.2608; Fig. 6e), trehalose showed a tendency to decrease the aggregates area (Area_Control_ = 1.282 ± 0.337 µm^2^/vg/cel; Area_Treatment_ = 0.719 ± 0.075 µm^2^/vg/cel; p = 0.1591; Fig. 6f, g), indicative of a reduced aggregates size. These observations reveal a protective effect of trehalose regarding neuropathologic features that characterize this lentiviral model of MJD.

## Discussion

In the present work, we show that administration of trehalose alleviates the phenotype of two mouse models of MJD, suggesting trehalose may be a potential pharmacological candidate for MJD.

It has been shown that trehalose acts as an autophagy inducer [14] and as a chemical chaperone preventing protein misfolding and aggregation [34]. These properties have pointed towards the use of trehalose for the treatment of protein aggregation disorders. Therapeutic effects of trehalose have been reported in a number of diseases using both in vitro and in vivo models, but not in MJD [35].

Before testing trehalose in vivo, we investigated whether trehalose would be able to activate autophagy in an in vitro model of MJD. This in vitro model was generated by expressing an expanded form of human ataxin-3 in mouse neuroblastoma cells [8]. We observed a time-dependent activation of autophagy by trehalose, with a remarkable effect after 24 h, 48 h and 72 h treatment at a concentration of 10 mM. The activation of autophagy parallels the decrease in the mutant ataxin-3 protein, showing that trehalose is effective in reducing mutant protein levels. These results are in accordance with previously described time-dependent effects of trehalose in reducing aggregated proteins through autophagy activation in neuronal cell models of Prions’s [15], Parkinson’s and Huntington’s diseases [14].

Based on the in vitro results, we pursued a study using an animal model expressing a truncated form of human ataxin-3 in Purkinje cells of the cerebellum [21]. The cerebellum is one of the most affected brain regions in MJD, being essential for motor coordination and balance [36]. Dysfunction and death of cerebellar Purkinje cells lead to degeneration of connected cells present in other cerebellar layers and consequently to cerebellar atrophy, which is particularly marked in this animal model [21]. These MJD transgenic mice display a severe cerebellar ataxic behaviour with pronounced impairments in coordination, balance and gait patterns since the 3^rd^ week of age [9, 11, 21]. When animals were administered with 2% trehalose we observed beneficial therapeutic effects in motor behaviour of the transgenic mouse model. This is particularly relevant considering the aggressive phenotype presented by these mice and the fact that trehalose administration started at 5 weeks of age, after the onset of the disease. Furthermore, we observed that trehalose effects on behaviour were associated with a reduction of the MJD-associated neuropathology. MJD transgenic mice receiving trehalose presented less atrophy of cerebellar layers, suggesting a prevention of neurodegeneration by trehalose. In agreement, an improvement of neuropathologic features was also observed in the lentiviral-based model, which expresses the full-length form of human ataxin-3. These observations are in accordance with those reported for other polyglutamine disorders. In Huntington’s disease, 2% trehalose treatment reduced foot-clasping posture and improved rotarod performance and footprint patterns of R6/2 transgenic mice [20]. These animals also displayed a reduction in striatum atrophy and in aggregate number. Similarly, treatment of SCA17 transgenic mice with 4% trehalose increased latency to fall in the rotarod and mediated amelioration of gait disturbances in the footprint analysis [37].

Neuronal intranuclear inclusions detected in the brain of patients with MJD are considered an important hallmark of the disease [3, 4, 38]. A reduction in the number of inclusions by trehalose has been reported in models of polyQ diseases and associated with improvements of the disease phenotype [15, 16, 19, 39], however this effect has not been consistently observed [37, 40, 41]. In a SCA17 mouse model, trehalose significantly improved animal’s behaviour, but had no significant effect in aggregate number [37]. Similarly, we did not detect differences in the number of aggregates of trehalose-treated animals. However, we observed a significant reduction in aggregates size after trehalose administration. The role of aggregation in the neuropathology of polyQ diseases remains controversial and the presence of mutant protein inclusions seems to not fully explain the toxicity seen in patients and mouse models [42, 43]. Even though aggregates fragments and oligomers have been considered toxic species, many studies suggest that the type of aggregates and its conformation are likely to play a more critical role in toxicity due to ability to differently sequester proteins and components, independently of their size [42–45]. Accordingly, we previously demonstrated that larger aggregates presented increased co-localization with caspase-3, suggesting an increased cytotoxicity compared to smaller aggregates [46].

Increasing amount of evidence show that trehalose exerts its neuroprotective effects by several mechanisms. Trehalose was detected in brain homogenates of mice treated with 2% trehalose in the drinking water [20, 47], which support the hypothesis of a direct effect in the brain, probably by inducing autophagy via the lysosomal-mediated TFEB activation [48–50]. By activating autophagy, trehalose seems to induce the clearance of unfolded proteins and oligomers, preventing or delaying the formation of larger inclusions [14]. In our experiments, we did not observe changes in autophagy markers after 2 weeks administration of 2% trehalose in the lentiviral-based model. This was not surprising, as one of the main challenges in the autophagy field is the lack of reliable tools to monitor autophagy activity in vivo. Autophagy is a highly dynamic, multi-step process and can be modulated at several steps [51] and the static analysis of LC3BII or p62 levels have limitations in capturing this dynamic process. For instance, a concomitant rise in both autophagosome formation rate and LC3-downstream degradation can show normal steady-state levels in LC3II protein despite increased autophagy activity. Thus, the use of autophagy markers in this study needs to be further complemented in other studies that estimate overall autophagic flux to allow a definitive interpretation of the observations, as stated in autophagy guidelines [52].

It is noteworthy that despite the presence of trehalase in the gut, orally administered trehalose has been proven to exert significant biological effects in mouse models of many different diseases, such as Parkinson’s, Alzheimer’s, muscular dystrophy, Huntington’s disease, ALS, prion’s disease, OPMD, obesity, hepatic steatosis, diabetes, or chronic ischemia [35, 53, 54]. Thus, we cannot exclude that trehalose effects might occur, or at least count with contribution of indirect effects in peripheral energy metabolism and inflammatory pathways [55, 56]. Therapeutic strategies that improves metabolism energy and inflammation, such as caloric restriction [22] and ibuprofen [57], are indeed effective in MJD mouse models. Interestingly, trehalose can also affect gut microbioma [58] , which raise the hypothesis that trehalose might interfere by modulating the microbiota-gut-brain axis. Further studies would be of utmost importance to clarify the mechanism of action of trehalose in MJD and other neurodegenerative disorders.

Another relevant aspect concerns the magnitude of the beneficial effects of trehalose. When compared to other molecules, the effects of trehalose in this MJD model seem to be less pronounced and less significant [22, 46]. This might be related to the high variability observed between animals, the low number of animals used (a major limitation of this study) and/or the use of a low dosage. Even though this 2% trehalose dose has been previously used in animal models [14, 17, 20, 59], administration of a higher dose of trehalose is well tolerated [37] and would likely lead to more pronounced effects in this severe animal model. Furthermore, initiating the treatment at an early stage would likely be advantageous, as it was shown for other drugs [60].

Nevertheless, our data does not support an effect comparable to our most robust approaches, namely gene silencing [9] or caloric restriction [22] or other promising strategies, such as antisense oligonucleotides [61, 62]. An interesting approach could be the use trehalose in combination with these therapies.

The major advantage supporting the use of trehalose in MJD is related to its safety. Acute and subchronic toxicological studies demonstrated that trehalose has no significant toxic effects in both animals and humans [63]. Despite being a sugar, trehalose has no adverse effects on metabolism [59, 63–65]. Some studies even reported a positive effect of trehalose against weight loss in mice [16, 20], which would constitute an advantage as MJD patients have decreased body mass index [66, 67]. A Phase 2 open label trial to access safety, tolerability and efficacy of trehalose in MJD patients is on-going.

In a phase 2 clinical trial to treat Oculopharyngeal Muscular Dystrophy (OPMD), trehalose was administered by intravenous injection, and despite the high dose used, no safety issues were identified [68]. More relevant, a post hoc analysis of the cold water, nectar and honey-thickened drinking tests showed that OPMD patients had a significant reduction in drinking time as compared to baseline (NCT02015481). This clinical study confirmed data from animal models [17] and brought expectations regarding the use of trehalose in protein-aggregation pathologies.

## Conclusions

In conclusion, this study shows that trehalose alleviates motor impairments and neuropathological features in MJD mouse models. Further studies would be important to clarify the mechanism of neuroprotection by trehalose in MJD, to determine the most effective dose and evaluate the potential of its association with other therapeutic approaches. Nevertheless, given the favorable safety profile of trehalose this molecule has potential to be used as a pharmacological drug for MJD, alone or in combination with other therapies.

## Supplementary information

**Additional file 1: Fig. S1.** Cellular activity of N2A cells expressing mutant ataxin-3 after treatment with Trehalose.

**Additional file 2:Fig. S2.** Uncropped western blot membranes of Fig. 1a.

**Additional file 3: Fig. S3.** Uncropped western blot membranes of Fig. 1c.

**Additional file 4: Fig. S4.** Transcript levels of mutant ataxin-3 and autophagy markers in neuro-2a cells expressing mutant ataxin-3 after 72 h treatment with Trehalose.

**Additional file 5: Fig. S5.** Body weight and general physical health of MJD transgenic mice treated with 2% Trehalose.

**Additional file 6: Fig. S6.** Mutant ataxin-3 levels in striatal lentiviral-based model of MJD treated with 2% Trehalose.

**Additional file 7: Fig. S7.** Western blot membranes of autophagy markers of the lentiviral-based MJD model.

## Data Availability

The datasets used and/or analysed during the current study are available from the corresponding author on reasonable request.

## Abbreviations

DARPP-32: Dopamine- and cAMP-regulated neuronal phosphoprotein
DTT: Dithiothreitol
HA: Hemagglutinin
LC3B: Microtubule-associated proteins 1A/1B light chain 3B
MJD: Machado–Joseph Disease
MutAtx-3: Mutant ataxin-3
PBS: Phosphate buffered saline
PFA: Paraformaldehyde
RIPA: Radioimmunoprecipitation assay
SDS: Sodium dodecyl sulfate
