# Supplementary figures and images for: Trehalose alleviates the phenotype of Machado–Joseph disease mouse models
Source: J Transl Med. 2020 Apr 9;18:161. doi: 10.1186/s12967-020-02302-2 (PMC7144062; doi:10.1186/s12967-020-02302-2)

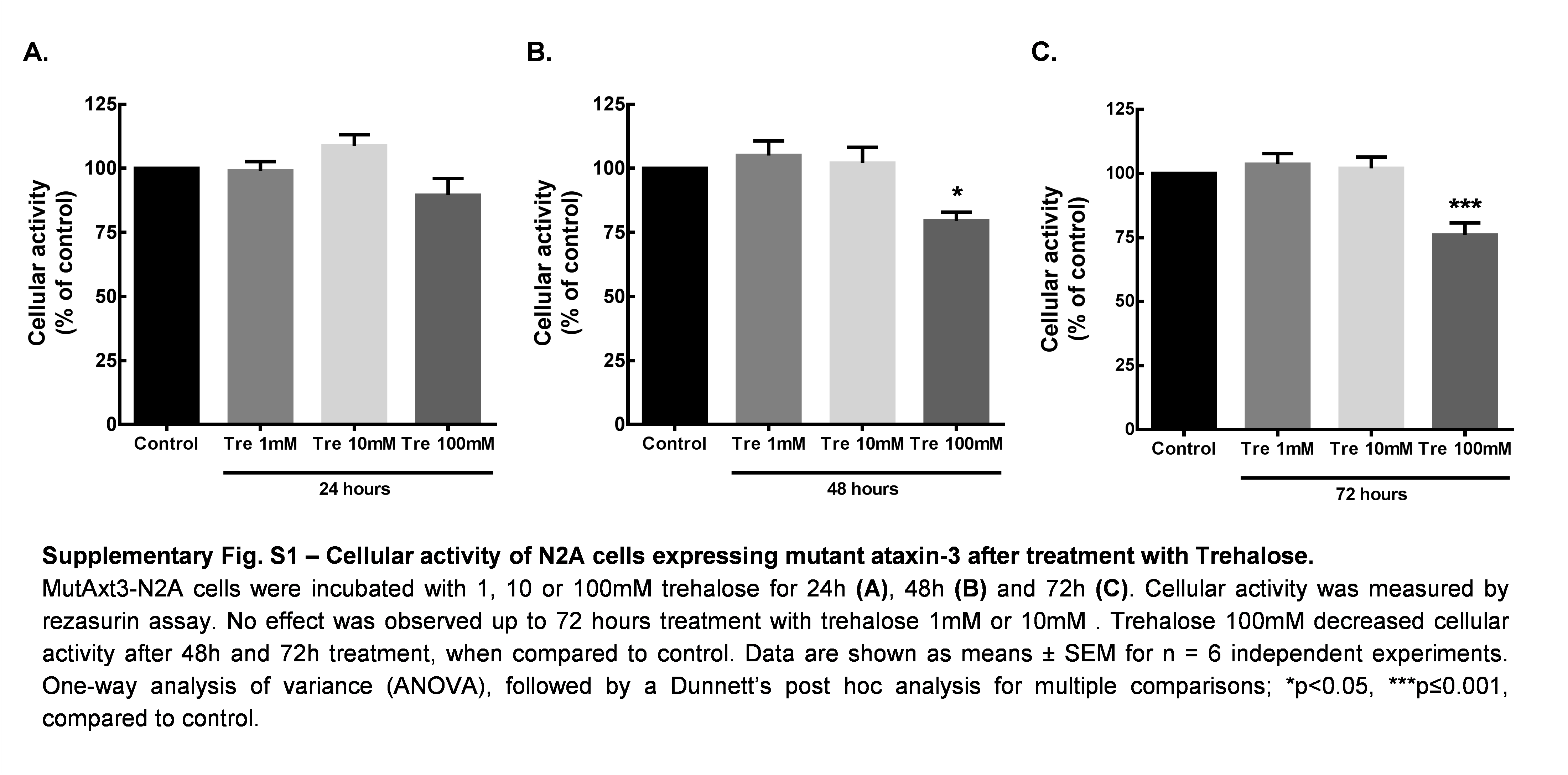

Supplement: Supplementary file 1 — Additional file 1: Fig. S1. Cellular activity of N2A cells expressing mutant ataxin-3 after treatment with Trehalose. [file 12967_2020_2302_MOESM1_ESM.tif]

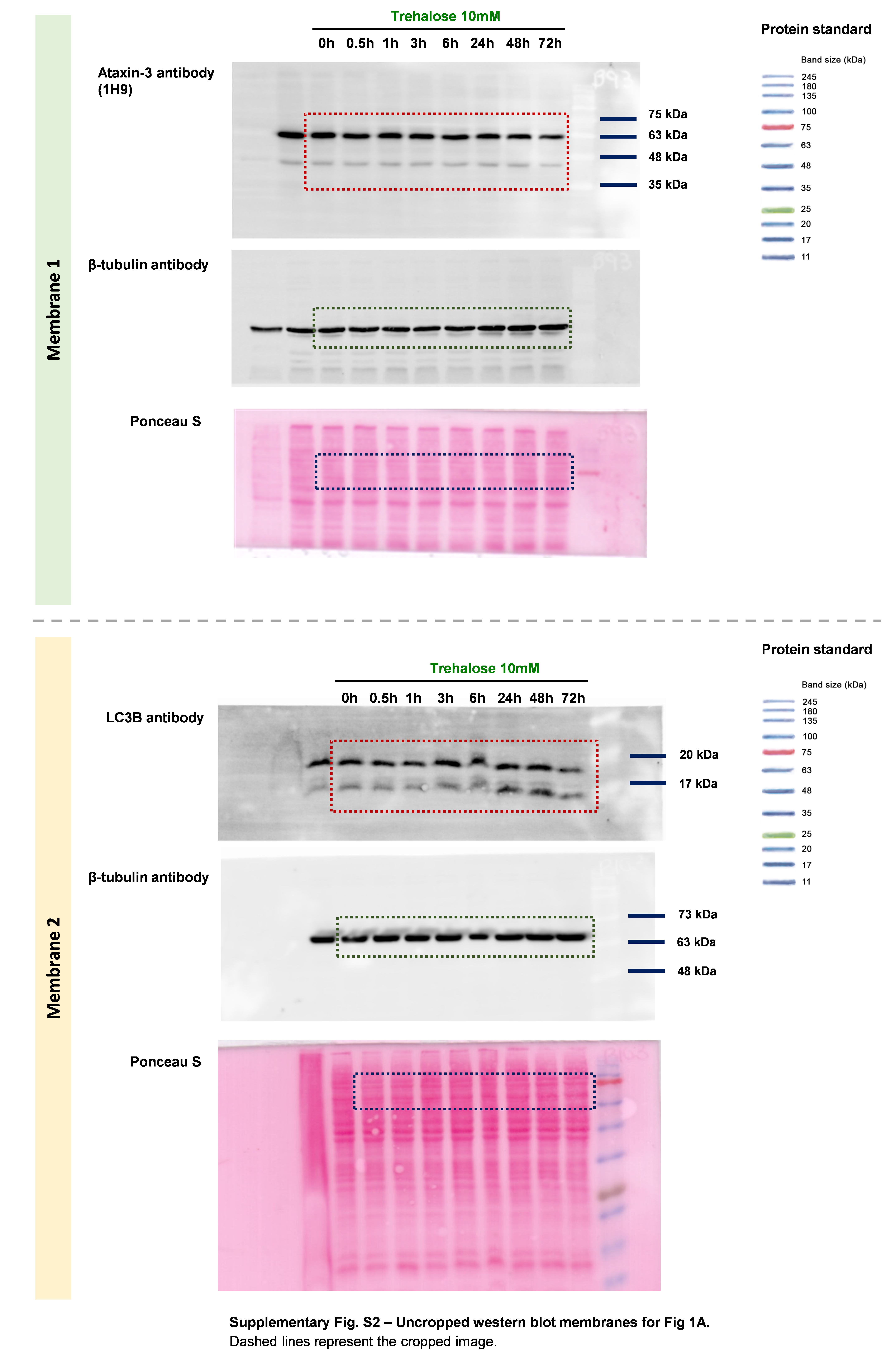

Supplement: Supplementary file 2 — Additional file 2:Fig. S2. Uncropped western blot membranes of Fig. 1a. [file 12967_2020_2302_MOESM2_ESM.tif]

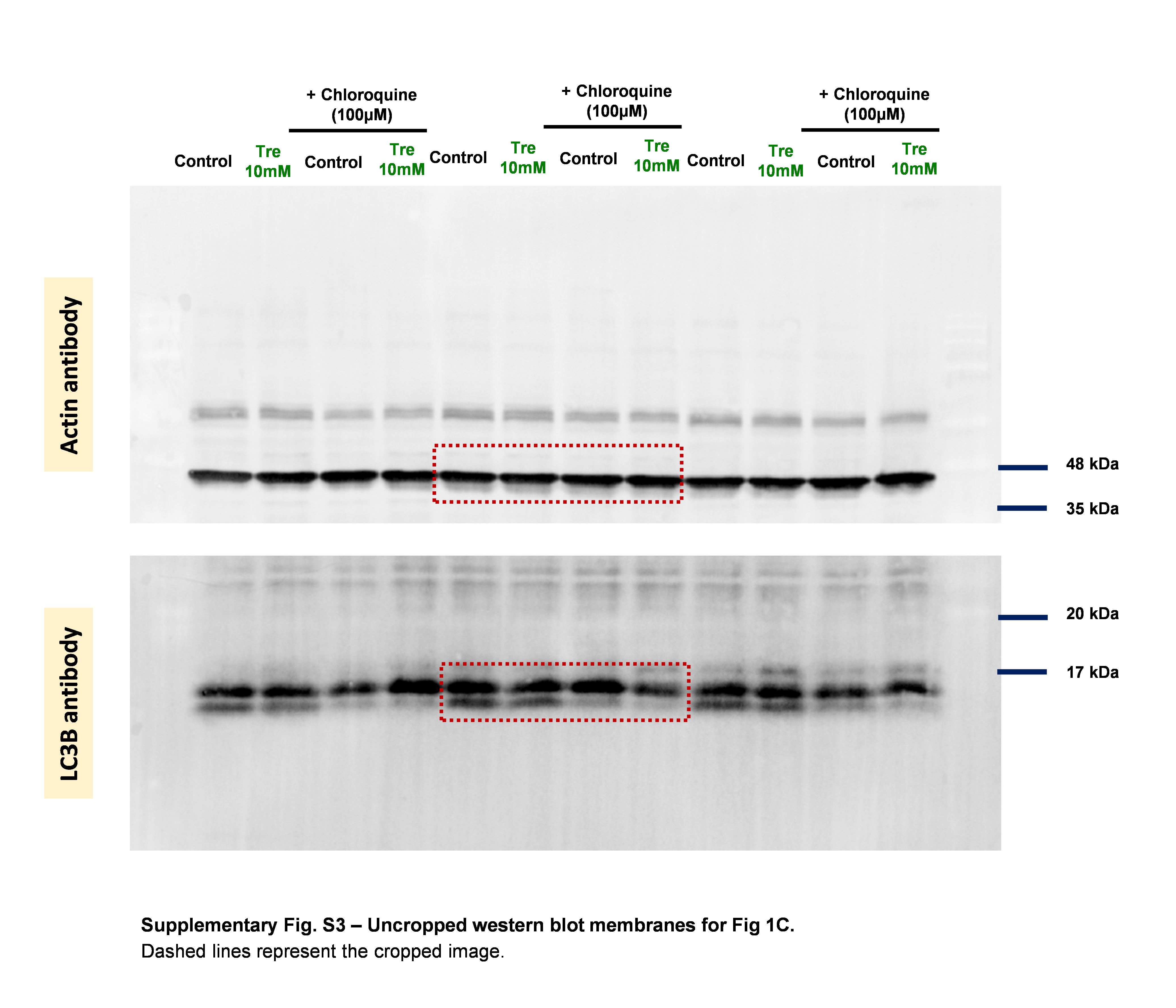

Supplement: Supplementary file 3 — Additional file 3: Fig. S3. Uncropped western blot membranes of Fig. 1c. [file 12967_2020_2302_MOESM3_ESM.tif]

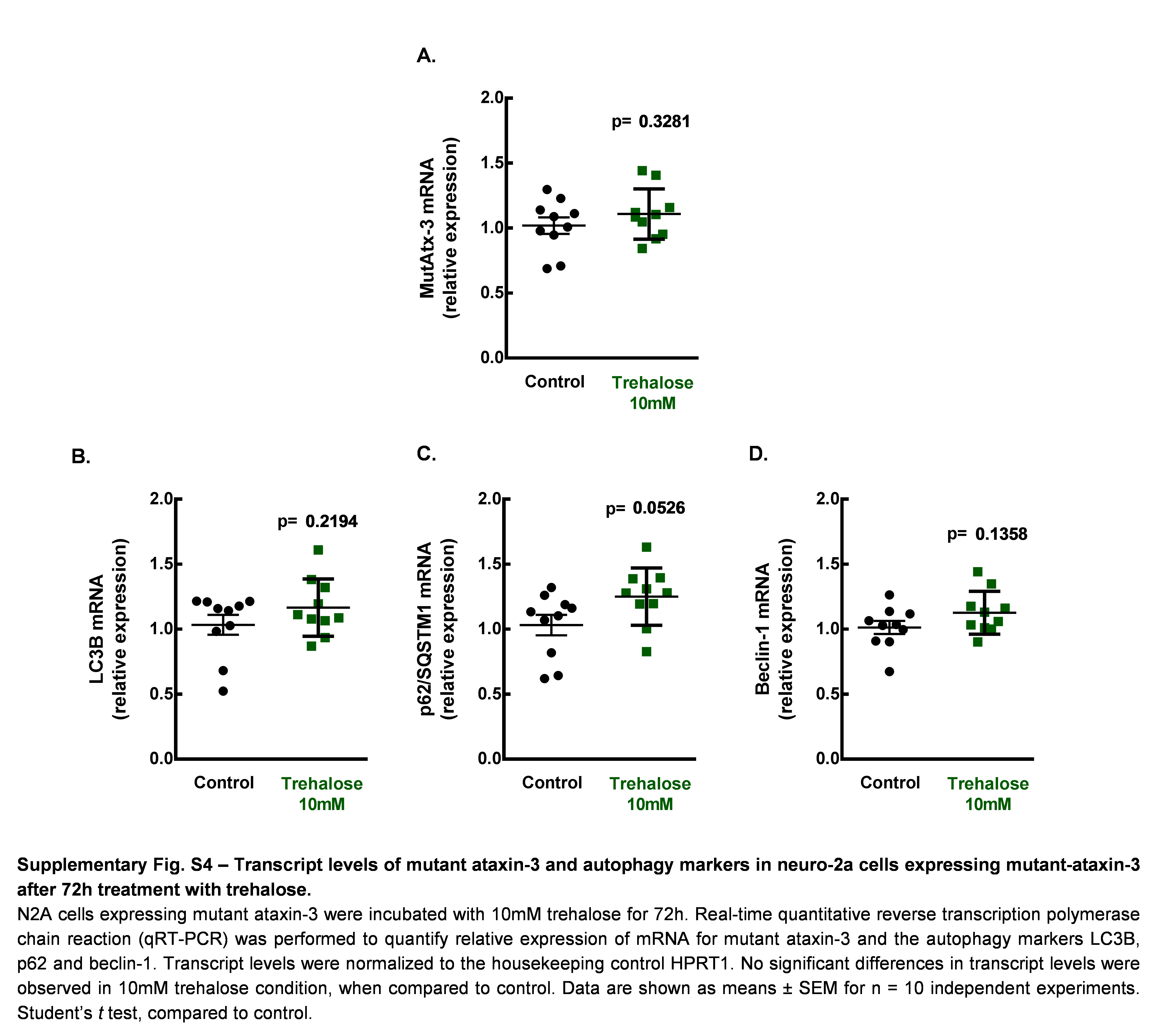

Supplement: Supplementary file 4 — Additional file 4: Fig. S4. Transcript levels of mutant ataxin-3 and autophagy markers in neuro-2a cells expressing mutant ataxin-3 after 72 h treatment with Trehalose. [file 12967_2020_2302_MOESM4_ESM.tif]

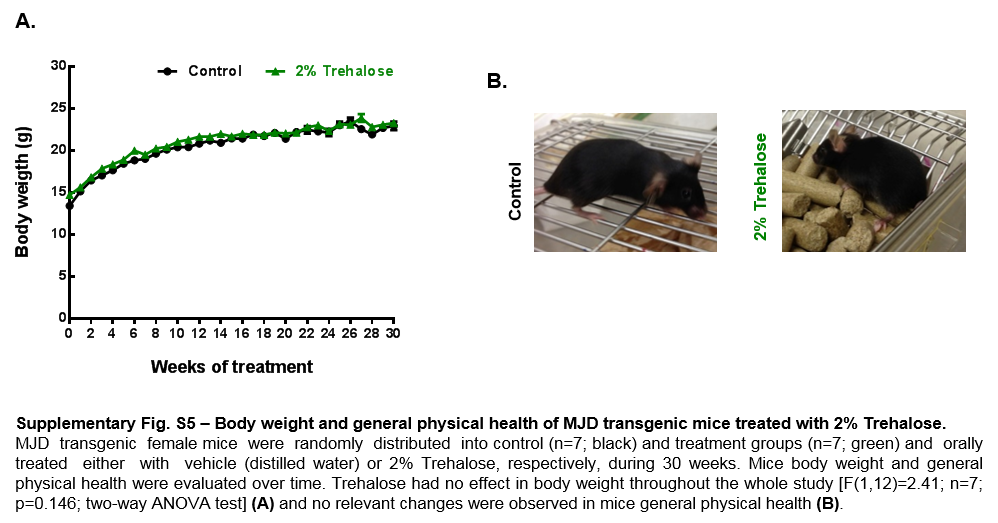

Supplement: Supplementary file 5 — Additional file 5: Fig. S5. Body weight and general physical health of MJD transgenic mice treated with 2% Trehalose. [file 12967_2020_2302_MOESM5_ESM.tif]

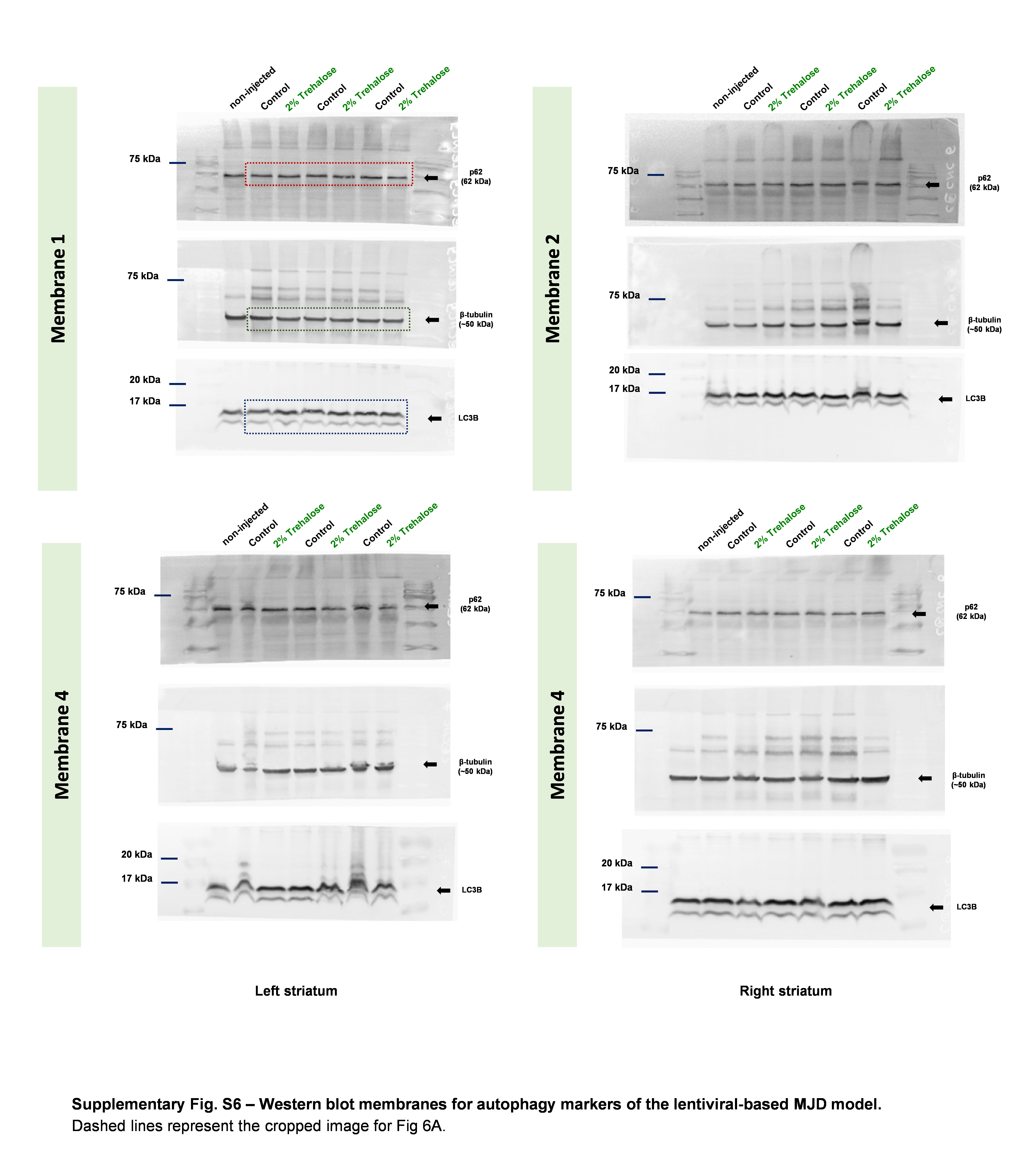

Supplement: Supplementary file 6 — Additional file 6: Fig. S6. Mutant ataxin-3 levels in striatal lentiviral-based model of MJD treated with 2% Trehalose. [file 12967_2020_2302_MOESM6_ESM.tif]

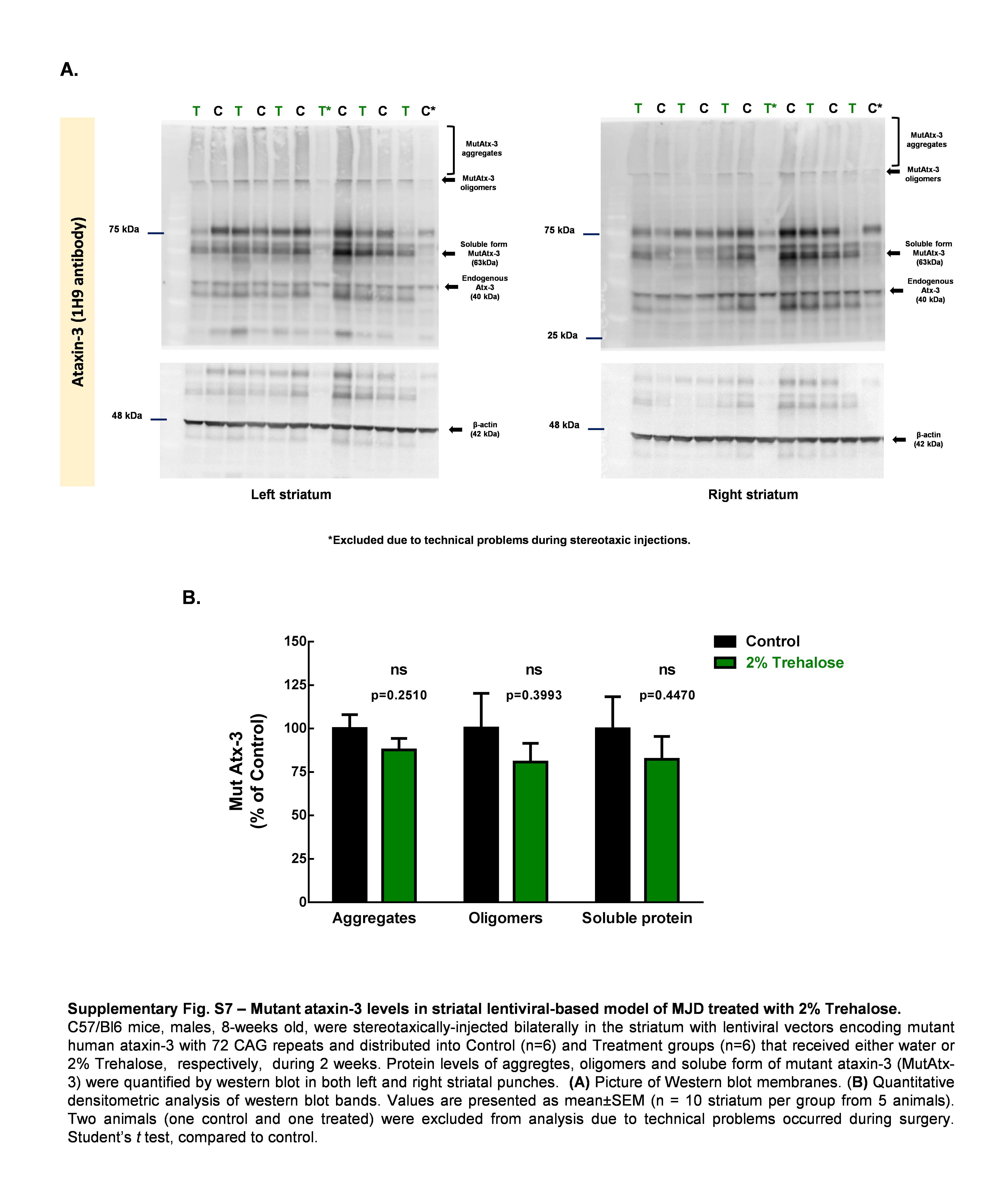

Supplement: Supplementary file 7 — Additional file 7: Fig. S7. Western blot membranes of autophagy markers of the lentiviral-based MJD model. [file 12967_2020_2302_MOESM7_ESM.tif]
